# Outcomes of lung transplantation surgery performed at night

**DOI:** 10.1016/j.xjon.2026.101827

**Published:** 2026-04-20

**Authors:** Sanne J.J. Langmuur, Jesse J. Duijghuisen, Merel E. Hellemons, Sanne E. Hoeks, Maarten ter Horst, Edris A.F. Mahtab

**Affiliations:** a Department of Cardiothoracic Surgery, Erasmus Medical Center, Rotterdam, The Netherlands; b Erasmus MC Transplant Institute, Erasmus Medical Center, Rotterdam, The Netherlands; c Department of Anaesthesiology, Erasmus Medical Center, Rotterdam, The Netherlands; d Department of Pulmonology, Erasmus Medical Center, Rotterdam, The Netherlands; e Department of Cardiothoracic Surgery, Leiden University Medical Center, Leiden, The Netherlands

**Keywords:** lung transplantation, time of surgery, night shift

## Abstract

**Objective:**

The logistics and planning involved in lung transplantation (LuTx) are complex. Recent advancements in LuTx surgery have introduced the possibility of deferring surgery to daytime hours and might reduce the burden on operating personnel and improve patient outcomes; however, this might interfere with the daytime program and could be labor-intensive and costly. Therefore, this study sought to investigate the effect of time of surgery on LuTx outcomes.

**Methods:**

Adult patients undergoing LuTx between 2010 and 2022 were included in this single-center retrospective cohort study. Patients were categorized as the night group if at least 1 hour of their operation occurred between 2 am and 6am. Otherwise, they were assigned to the day group. The primary outcome for this study was intensive care unit length of stay. Secondary outcomes included 30-day and 1-year survival, bronchiolitis-obliterans syndrome-free survival, time to extubation, rethoracotomy, need for extracorporeal circulatory support, blood loss and transfusion parameters, and ischemic, implant, and operating times. Both unadjusted comparisons and multivariable regression analyses were performed.

**Results:**

In total, 227 patients (day: n = 142, night: n = 85) were included in this study. After multivariable regression, no differences were found in survival, ICU and hospital length of stay, time to extubation, or operation duration. Additionally, no significant differences were found for bronchiolitis-obliterans free-survival, ischemic and implant times, use of extracorporeal circulation, wound complications, rethoracotomies, and the majority of blood loss and transfusion data in the unadjusted analysis.

**Conclusions:**

No differences were found between the day and night group regarding survival, length of stay, time to extubation and other (post-)operative outcomes. These findings indicate that performing LuTx surgery at night is safe, provided that this complex care is well-organized and delivered by a dedicated team.

## Graphical Abstract

**Figure:**
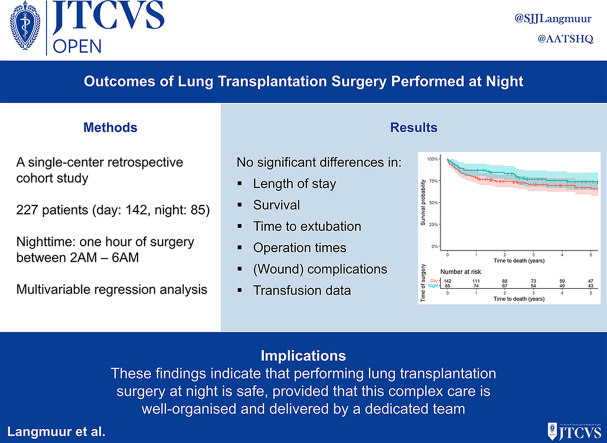

**Figure:**
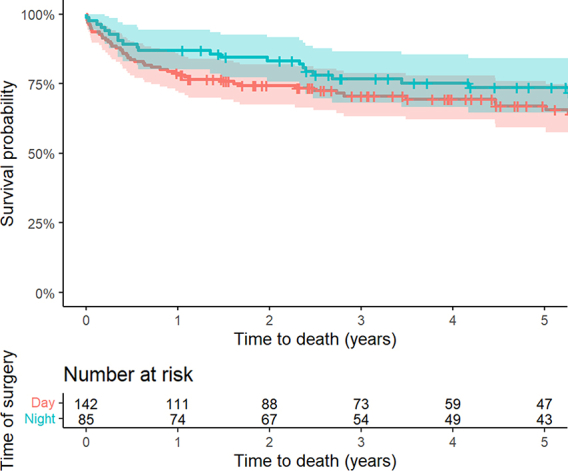
Survival curves for lung transplant during the day versus night.

**Central Message**

Lung transplantation performed at night has similar outcomes as daytime surgery. This indicates it is safe, provided that this complex care is well-organized and delivered by a dedicated team.

**Perspective**

Recent advancements in lung transplantation allow deferring surgery to the daytime but can and be resource-intensive and potentially interfere with elective programs. Therefore, outcomes of patients transplanted at night versus during the day were compared. No significant differences were found in survival, length of stay, time to extubation, operation times, (wound) complications, or transfusion data.

The logistics and planning involved in lung transplantation (LuTx) surgery are complex. Historically, the timing of this type of surgery has been dictated by the availability of a donor organ and the minimalization of cold ischemic times of the donor lungs. As a consequence, many LuTx surgeries take place outside of regular hours.

In general, nighttime surgery is associated with a greater occurrence of adverse events and increased mortality compared with daytime surgery in a range of different procedures.^1^, ^2^, ^3^ This could be the result of factors influenced by the circadian rhythm of the patient, human factors such as fatigue on the part of the operating personnel, and the unavailability of sufficient qualified staff during the night.^2^^,^^4^^,^^5^ However, the potential advantages of nighttime surgery include having reduced competition for operating rooms and fewer scheduling conflicts with the elective surgery program. In the literature on LuTx, evidence regarding the potential effect of timing of surgery is conflicting, and there is no uniform definition of nighttime surgery.^6^, ^7^, ^8^, ^9^

Recent advancements in LuTx surgery, including ex vivo lung perfusion and extended storage at 10 °C, have introduced the possibility of deferring surgery to daytime hours.^10^, ^11^, ^12^ However, this may interfere with the daytime logistics of the operating room, and some of these techniques are labor-intensive and costly. The availability of this option prompts the question whether LuTx should exclusively occur during the day, considering patient outcomes and the burden on the operating personnel. Therefore, this study sought to investigate the effect of time of surgery on LuTx outcomes.

## Methods

This retrospective cohort study included adult patients undergoing bilateral LuTx at the Erasmus Medical Center between 2010 and 2022. Patients undergoing unilateral LuTx, redo LuTx, and LuTx after ex vivo lung perfusion were excluded for this analysis. Patients were categorized as the night group if at least 1 hour of their operation (defined as skin incision to skin closure) occurred between 2 am and 6am. Otherwise, they were assigned to the day group. If the time of operation was unknown, the patient was excluded from analysis. Nighttime surgery lacks a universally accepted definition in the existing literature, with different studies reporting different time frames.^1^, ^2^, ^3^, ^4^, ^5^, ^6^, ^7^, ^8^, ^9^ In consultation with experienced staff members, the 2- to 6-am time window was considered as the most appropriate window, taking into account circadian rhythms and regular elective operating schedule in our center. This study adhered to the Strengthening the Reporting of Observational Studies in Epidemiology guidelines.^13^

### Study Outcomes

The primary outcome for this study was intensive care unit (ICU) length of stay (LOS). Secondary outcomes included 30-day and 1-year survival, bronchiolitis obliterans syndrome (BOS)-free survival, time to extubation, rethoracotomy, intraoperative need for extracorporeal circulatory support (ECC) (defined as either extracorporeal membrane oxygenation [ECMO] or cardiopulmonary bypass [CPB]), blood loss and transfusion parameters, and ischemic, implant, and operating times.

### Data Collection

Data were collected from the electronic medical records. On the basis of clinical LuTx care, patients provided informed consent for data collection. Use of these data was approved by the medical ethical committee of the Erasmus Medical Center (MEC-2024-0321, date of approval July 25, 2024). Data on operating times, blood loss, and transfusion were extracted from the operation registration.

Implant time was defined as the time of the first incision to the time of recirculation of the donor lung. Operation duration was calculated as time of first incision to time of skin closure. Blood loss was registered as the amount of blood collected by drapes and gauzes that were weighed intraoperatively and the volume collected by the cell saver (fluids other than blood excluded). Wound-related complications were collected as registered in the patient’s electronic medical record. They were considered superficial if they concerned only the soft tissues or steel wire removal and deep if mediastinitis or sternal dehiscence or dislocation was present or rib refixation was required. Patients were considered as high urgency (HU) status after unanimous decision of the national HU committee (before 2014) or if they had a lung allocation score ≥50 (after 2014).

### Organ Allocation and Surgical Procedure

Donor lungs were allocated through Eurotransplant. The time of organ retrieval, as determined by the organ donation coordinator based on factors such as hospital logistics and family preference, dictated the time of surgery. Donor lungs were retrieved by the surgeons from our hospital and were transported to the recipient hospital in PERFADEX solution (XVIVO) on static cold storage with ice. The surgical procedure was described in more detail previously.^14^, ^15^, ^16^ In short, a standardized local anesthesia protocol was followed. Patients received a central venous catheter via the jugular vein and invasive arterial blood pressure catheter in the radial artery and were intubated with a double-lumen tube. Cardiac function was monitored using transesophageal ultrasonongraphy. For patients with severe pulmonary hypertension, ECMO was installed before induction or before the start of surgery. Earlier in the study period, patients were operated on using the clamshell incision, but this gradually shifted toward the bilateral anterolateral thoracotomy.^16^ ECC was placed centrally or peripherally, respectively. Other time-related changes were the transition from intraoperative CPB use to ECMO and the introduction of a rotational thromboelastometry–guided protocol for transfusion management.^14^ Preoperative, intraoperative, and postoperative management of these patients was strictly regulated in local protocols written by all care providers involved in the LuTx care.

### Statistical Analysis

All statistical analyses were performed using R (R Foundation for Statistical Computing; version 4.4.0). Categorical variables were reported as number (%). Normally distributed continuous variables were described as mean ± standard deviation and non-normally distributed variables as median with interquartile range. Normality was tested using QQ-plots, density plots, and Shapiro-Wilk tests. Student *t* tests, Mann-Whitney *U* tests, or χ^2^ tests were used as appropriate. No correction for multiple testing was performed.

For the primary outcome, ICU LOS, multivariable regression was used to adjust for possible confounding. The model included age, sex, BMI, HU status, LuTx indication, intraoperative ECC use, and operative technique. These variables were selected on the basis of existing literature and clinical expertise. A similar analysis was performed for secondary outcomes ICU and hospital LOS, time to extubation, and operation duration. Poisson regression was used for the LOS at the ICU and hospital and time to extubation. If overdispersion was present, quasi-Poisson was used instead. Linear regression was used for the operation duration outcome. Model assumptions were checked by plotting the residuals. The multicollinearity assumption was assessed by measuring the variance inflation factor, which should have a value ≤5.

BOS-free survival was a composite outcome of BOS or death, whichever event occurred first. Both 30-day and 1-year survival were compared using χ^2^ tests, and late survival was calculated and visualized using the Kaplan-Meier method and compared with the log-rank test (in case the proportional hazards assumption was not violated). A Cox regression model was built correcting for the same set of potential confounders, to calculate the hazard ratio of mortality between the groups.

A sensitivity analysis was added to make the comparison between this study's outcomes and existing literature more feasible. This sensitivity analysis was performed in a similar fashion as the regression and survival analyses of the main results, but with a different time frame. The night group was defined as patients with a time of skin incision between 7 pm and 5 am. All other patients were included in the day group for this sensitivity analysis.

## Results

**Figure 1:**
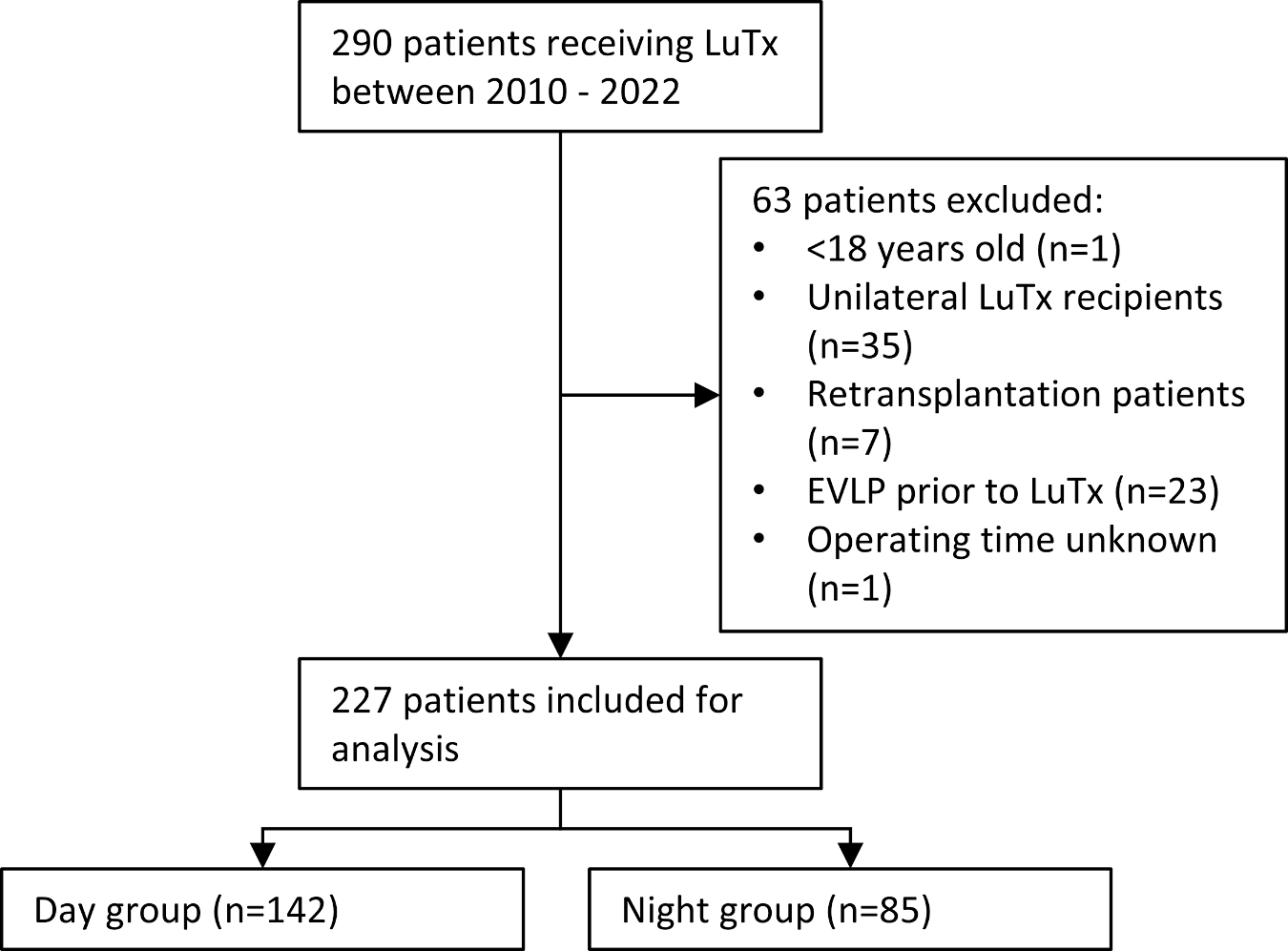
Flow diagram of the inclusion and exclusion criteria. *LuTx*, Lung transplantation; *EVLP*, ex vivo lung perfusion.

In total, 227 patients were included in this study (142 patients in the day group and 85 patients in the night group) (Figure 1). Baseline characteristics are described in Table 1. No statistically significant differences were found between the day and night group.

**Table 1:** Baseline characteristics

Characteristic	Day (n = 142)	Night (n = 85)	*P* value
Age, y	58 (50-62)	56 (50-61)	.20
Male sex	75 (53%)	41 (48%)	.60
BMI	24 (21-27)	24 (22-27)	.92
LuTx indication			.08
COPD	59 (41%)	26 (30%)	
ILD	58 (41%)	44 (52%)	
CF	18 (13%)	15 (18%)	
PH	6 (4%)	0 (0%)	
BE	1 (1%)	0 (0%)	
HU status	37 (26%)	27 (32%)	.44
Waiting list time, y	0.8 (0.2-1.8)	1.3 (0.4-2.7)	.07
Hospitalization pre-Tx	43 (30%)	26 (31%)	1.00
ICU pre-Tx	19 (13%)	9 (11%)	.68
ECMO pre-Tx			.77
VV	6 (4%)	5 (6%)	
VA	5 (3%)	2 (2%)	
VAV	1 (1%)	0 (0%)	
No ECMO	130 (92%)	78 (92%)	

Continuous variables are presented as mean ± standard deviation or median (interquartile range). Categorical variables are presented as n (%). *BMI*, Body mass index; *LuTx*, lung transplantation; *COPD*, chronic obstructive pulmonary disease; *ILD*, interstitial lung disease; *CF*, cystic fibrosis; *PH*, pulmonary hypertension; *BE*, bronchiectasis; *HU*, high urgency; *ICU*, intensive care unit; *Tx*, transplantation; *ECMO*, extracorporeal membrane oxygenation; *VV*, venovenous; *VA*, venoarterial; *VAV*, venoarterial-venous.

### Postoperative Outcomes

In the unadjusted analysis, median ICU LOS was 10 (4-37) days for the day group and 8 (4-30) days in the night group (*P* = .51) (Table 2). Hospital LOS was 33 (25-64) days and 40 (28-73) days, respectively (*P* = .17). No statistically significant differences were found between groups for the need for a rethoracotomy, the time to extubation post-LuTx, or wound-related complications (Table 2).

**Table 2:** Postoperative outcomes

Outcome	Day (n = 142)	Night (n = 85)	*P* value
LOS ICU, d	10 (4-37)	8 (4-30)	.51
LOS hospital, d	33 (25-64)	40 (28-73)	.17
Rethoracotomy	34 (24%)	21 (25%)	1.00
Hemorrhagic	27 (19%)	19 (22%)	
Time to extubation, d	2 (1-9)	1 (1-4)	.19
Wound complications	40 (28%)	27 (32%)	.47
Superficial	24 (17%)	22 (26%)	
Deep	20 (14%)	8 (9%)	
30-d survival	133 (94%)	83 (98%)	.30
1-y survival	111 (78%)	74 (87%)	.14
3-y BOS-free survival	63% (95% CI, 55-72%)	70% (95% CI, 61-81%)	.3∗

Continuous variables are presented as mean ± standard deviation or median (interquartile range). Categorical variables are presented as n (%). *LOS*, Length of stay; *ICU*, intensive care unit; *BOS*, bronchiolitis obliterans syndrome.

∗ Log-rank *P* value.

**Figure 2:**
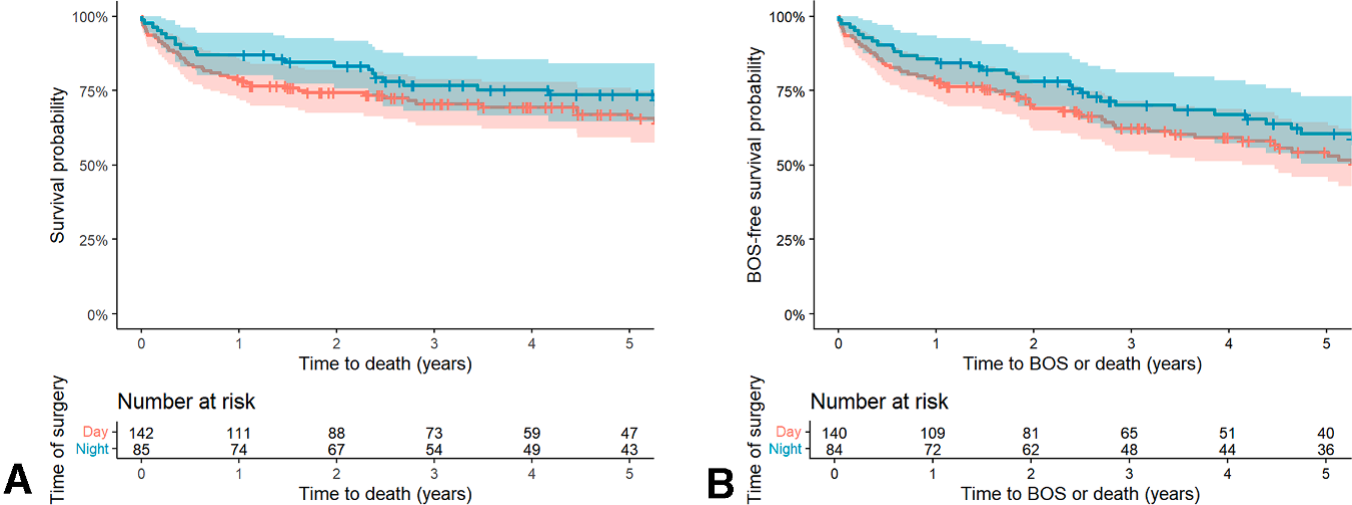
Kaplan-Meier plot with 95% CIs in the day versus night group for survival probability (A) and BOS-free survival probability (B). Log-rank test: *P* = .3 and *P* = .3. *BOS*, Bronchiolitis obliterans syndrome.

Thirty-day survival was 94% in the day group and 98% in the night group (*P* = .30), and 1-year survival was 78% versus 87% (*P* = .14) (Table 2). No patients were lost to follow-up within the first year. Three years posttransplantation, BOS had occurred in 10 (7%) versus 8 (9%) of patients, leading to a 3-year BOS-free survival of 63% (95% CI, 55-72%) versus 70% (95% CI, 61-81%). The Kaplan-Meier plots are shown in Figure 2. No significant differences were found between groups according to the log-rank test for both the survival curves (*P* = .3) and BOS-free survival curves (*P* = .3).

### Multivariable Analysis

No statistically significant differences were found between the day versus night groups for both the univariable and multivariable regression analyses for LOS in the ICU and hospital, time to extubation, or the operation duration (Table 3). Multivariable Cox regression, used to correct for age, sex, BMI, HU status, indication, intraoperative ECC use, and incision type, provided a hazard ratio of 0.85 (95% CI, 0.85-1.18, *P* = .49) for patients operated during the night.

**Table 3:** Unadjusted (univariable model) and adjusted (multivariable model) estimates for the effect of time of surgery (operating during the day as reference) on the outcome

Outcome	Univariable		Multivariable	
	β/IRR (95% CI)	*P* value	β/IRR (95% CI)	*P* value
LOS ICU, d∗	0.94 (0.63-1.36)	.74	0.88 (0.60-1.28)	.52
LOS hospital, d∗	1.01 (0.80-1.27)	.92	0.98 (0.78-1.22)	.83
Time to extubation, d∗	0.87 (0.46-1.57)	.65	0.76 (0.41-1.37)	.38
Operation duration (minutes, log transformed)†	0.03 (−0.05 to 0.11)	.45	0.01 (−0.06 to 0.08)	.76

In the multivariable model, outcomes were corrected for age, sex, body mass index, high urgency status, lung transplantation indication, intraoperative extracorporeal circulation use, and operative technique. *IRR*, Incidence rate ratio; *LOS*, length of stay; *ICU*, intensive care unit.

∗ Quasi-Poisson regression with incidence rate ratio.

† Linear regression with β (transformation in first column).

### Operative Outcomes

Operative outcomes can be found in Table 4. No statistically significant differences were found in ischemic (day: 416 [358-491] minutes vs night: 408 [372-478] minutes, *P* = .74) or implant times (320 [277-369] minutes vs 328 [280-394] minutes, *P* = .49), or operation duration (404 [350-488] minutes vs 406 [355-481] minutes, *P* = .69) (Table 4). The bilateral anterolateral thoracotomy was used more often in the day group as compared with the night group, in which the majority of the patients was operated through a clamshell incision (anterolateral thoracotomy: 80 [56%] versus 33 [39%], *P* = .02). ECC was used in 46% of patients in the day group and 52% in the night group (*P* = .46). Differences were not statistically significant, but the night group tended to use more CPB (36% vs 50%, *P* = .08), whereas the day group used more ECMO (venoarterial ECMO: 7% vs 1%, *P* = .08); venovenous ECMO: 3% vs 1%, *P* = .07).

**Table 4:** Operative characteristics

Outcome	Day (n = 142)	Night (n = 85)	*P* value
Ischemic time, min			
First lung	270 (238-325)	285 (239-319)	.97
Second lung	416 (358-491)	408 (372-478)	.74
Implant time, min			
First lung	173 (149-199)	184 (146-223)	.20
Second lung	320 (277-369)	328 (280-394)	.49
Operation duration, min	404 (350-488)	406 (355-481)	.69
Anterolateral thoracotomy	80 (56%)	33 (39%)	.02
Intraoperative ECC	65 (46%)	44 (52%)	.46
CPB	52 (36%)	42 (50%)	.08
VA-ECMO	10 (7%)	1 (1%)	.07
VV-ECMO	5 (3%)	1 (1%)	
Blood loss, mL	2345 (1355-4380)	2270 (1050-5418)	.93
Cellsaver blood return	700 (380-1450)	860 (325-1988)	.48
Erythrocytes, units	2 (0-7)	4 (1-8)	.19
Platelet concentrate, units	0 (0-1)	1 (0-2)	.03
Plasma volume, mL	800 (0-1400)	1200 (400-2000)	.08

Continuous variables are presented as mean ± standard deviation or median (interquartile range). Categorical variables are presented as n (%). *ECC*, Extracorporeal circulation; *CPB*, cardiopulmonary bypass; *VA*, venoarterial; *ECMO*, extracorporeal membrane oxygenation; *VV*, venovenous.

No significant differences were present in the majority of blood loss and transfusion data, with the exception of platelet concentrate transfusion, which was significantly lower in the day group (0 [0-1] vs 1 [0-2], *P* = .03) (Table 4). The day group received 2 (0-7) units of erythrocytes and 800 (0-1400) mL of plasma and the night group received 4 (1-8) units (*P* = .19) and 1200 (400-2000) mL (*P* = .08).

### Sensitivity Analysis

In the sensitivity analysis, the night group was defined as time of skin incision in the recipient between 7 pm and 5 am. In total, 19 patients (8%) changed groups with the new time frame definition, as compared with the definition in the original analysis in this study. No significant baseline differences were present (Table E1). No significant differences were found between groups for ICU LOS, hospital LOS, and time to extubation, in both uni- and multivariable analysis. Operation duration was significantly in favor of the night group after uni- and multivariable analysis (adjusted β −0.08 (95% CI −0.15 to −0.02), *P* = .01) (Table E2). Multivariable Cox regression provided a hazard ratio of 0.73 (95% CI, 0.45-1.16, *P* = .22).

## Discussion

This study sought to investigate the effect of time of surgery on LuTx outcomes. No significant differences were found between the day and the night groups regarding LOS in the ICU and hospital, survival, time to extubation, or operation duration, both in unadjusted and adjusted analyses. In addition, no relevant statistically significant differences were found for other operative or postoperative outcomes, such as blood loss, rethoracotomies, wound complications, or BOS-free survival.

The current study examined a broad range of outcome measures, including variables such as blood loss, transfusion data, and wound complications, which were not reported in previous research. Across all outcomes, no relevant significant differences were observed between the day and night groups in our study. Previous studies on this topic have reported inconsistent findings.

A large retrospective dataset study from the United States from 2011 did not show any clinically relevant differences regarding survival or postoperative complications.^6^ Moreover, a recent, large United Network for Organ Sharing database study did not find an effect of time of day of LuTx on short- or long-term survival when using a binary categorization.^9^ However, these authors did demonstrate an association between favorable short-term survival and certain times during the day when doing a continuous-time analysis.^9^ They did not find any relevant differences in secondary outcomes such as ECMO within 72 hours or airway dehiscence in their unadjusted analysis.^9^ On the contrary, 2 other, smaller retrospective cohort studies have found a worse outcome when lung transplantation was performed after hours.^7^^,^^8^ The authors of one study found a greater rate of primary graft dysfunction when the donor lungs were reperfused between 4 and 8 am.^8^ However, they did not find any differences in surrogate markers of human fatigue, such as operation duration or warm ischemic time, which is similar to our findings. The authors suggested the difference they found was attributable to internal circadian desynchrony between donor and recipient after organ preservation, which they then tested in a mice model.^8^ In another study, from the United States, the authors found a greater risk of major postoperative events and decreased 5-year overall and BOS-free survival when the time of incision was between 6 pm and 5 am.^7^ Costs were similar between the groups operated during the night versus the day, but they did not intentionally delay the LuTx overnight using a preservation device.^7^ In a recent study by Kim and colleagues,^12^ lung transplants, which would have been performed during the night, were postponed until the following morning using standard 4 °C cold static storage. In a propensity score–matched analysis, the authors found similar short- and long-term postoperative outcomes such as primary graft dysfunction, LOS, and mortality, suggesting this might also be a safe alternative workflow.

In the unadjusted analysis of our study, the only statistically significant difference was an increased use of platelet concentrate from median 0 to 1 units. A similar trend was observed for units of erythrocytes and plasma volume that were transfused, even though this did not reach statistical significance. However, because this was likely caused by the greater number of clamshell thoracotomies and greater use of CPB in the night group, this finding can probably be considered negligible. Previous studies did not report on these blood loss and transfusion outcomes.

One explanation for the fact that we found similar results in the day and night group is that, within the complex chain of care that lung transplantation comprises, the timing of the surgery may not be the most critical factor. Specialized care for patients who are undergoing lung transplant does not end in the operating room; it continues, especially during the critical first hours in the ICU. Lung transplantation is performed by a dedicated and multidisciplinary team of specialists, each contributing to the patient's overall outcome. The outcomes of LuTx are also dependent on preoperative care, donor selection, postoperative care on the ICU and the ward, and long-term patient follow-up. In addition, in our center all members of the operating team, including the surgeons, anesthesiologists, perfusionists, and operating room nurses, are working in shifts that last 8 to a maximum of 24 hours, which means a LuTx will usually start with a relatively fresh team. Moreover, at least 1 experienced, dedicated LuTx surgeon was present in the large majority of these cases, and the surgeries were usually performed by 2 staff members during the night as well as the day. In our center, the surgical, anesthesia, and operating room scrub nurse teams are dedicated to cardiothoracic surgery and not planned for general surgery (emergency) procedures in the same shift. If members of the operating team worked during the night, they were free from clinical duties the next day. It may well be that the LuTx is so well-organized that the timing of surgery has little impact on the outcome.

One other explanation for the inconsistent findings between different studies could be that nighttime surgery lacks a universally accepted definition. A recent meta-analysis investigating a range of different surgeries revealed notable variability in how after-hours surgery was defined.^2^ This was also reflected by the existing literature on the timing of LuTx, because various definitions were used in these studies. The definition of after-hours surgery was institution-dependent, on the basis of the hospital's logistics and on the authors’ point of view. In our study, we focused on a time frame most affected by human factors. After consulting experienced staff members, we believed that this was when the LuTx was performed between 2 am and 6 am for a substantial part, so at least 1 hour. This choice aligned with findings from a recent circadian rhythm study involving 2602 first-year physicians from the United States, where 5 am emerged as the most challenging time of day.^17^ Furthermore, in their recent study, Choi and colleagues^9^ did not find any significant differences when they applied a binary categorization but did find an association when using continuous-time analyses. Because the latter was not possible in the current study, the choice of time frame may also have influenced our findings.

To explore the influence of our choice of time frame on the study's outcomes, a sensitivity analysis was performed using a time frame that was more similar to some of the studies that did find some differences, mainly in favor of their day group. However, in our sensitivity analysis, differences remained not significantly different between groups after multivariable regression. Operation duration was found to be shorter in the night group.

### Strengths and Limitations

This study is subject to several limitations. First, its sample size was relatively small. Therefore, it is important that these results are interpreted with some caution, because this increases the chance of a type II error if the study was underpowered for certain outcomes. Moreover, late survival data were only reported in the Kaplan-Meier curves, because sample size became even smaller for the long-term follow-up. However, this sample size did allow us to go into detail on certain outcomes such as blood loss and transfusion data or wound complications, which were not previously reported. Nonetheless, outcomes or burden on personnel may differ in greater-volume transplant centers. Second, some selection bias could have been present in this study. For complex cases, such as recipients with pulmonary hypertension, the team may have been more likely to proceed with LuTx during the day when more resources and specialized staff were available. However, baseline characteristics were not significantly different, and we used multivariable regression analysis to correct for possible confounding, including urgency status, but residual confounding cannot fully be excluded. Furthermore, no correction for multiple testing was applied for this study, because the relatively small sample size already limited power and additional adjustment would have further increased the risk of a type II error. Nevertheless, the study provides exploratory insight into outcomes that have not previously been described. Lastly, no data on primary graft dysfunction were available in this study. However, time to extubation is known to correlate well with primary graft dysfunction and other outcomes, which is why it was used as a proxy in this study.^18^

### Clinical Perspective

On the basis of our findings and existing literature, we conclude that time of surgery does probably not play an important role for patient outcomes in LuTx, as long as nighttime surgery is performed responsibly and the care for these complex patients is optimized at any time of day. It should therefore be up to the individual centers to decide which logistics are most suitable for them. On the one hand, performing surgery during the day may be beneficial for the health and performance of the operating teams themselves. On the other hand, it is also advantageous to avoid disruptions to the elective surgery program.

## Conclusions

In conclusion, this study found no differences in operative and postoperative outcomes between patients undergoing LuTx surgery during the day versus the night. This suggests that performing LuTx surgery at night is safe, provided that this complex care is well-organized and delivered by a dedicated team. Therefore, the decision to perform LuTx surgery at night or to postpone it to the next day should be left to individual centers on the basis of what aligns best with their priorities and logistical capabilities to provide the best care for their patients.

## Conflict of Interest Statement

The authors reported no conflicts of interest.

The *Journal* policy requires editors and reviewers to disclose conflicts of interest and to decline handling or reviewing manuscripts for which they may have a conflict of interest. The editors and reviewers of this article have no conflicts of interest.

## Figures and Tables

**Table E1:** Baseline characteristics for the sensitivity analysis

Characteristic	Day (n = 141)	Night (n = 86)	*P* value
Age, y	57 (50-62)	57 (51-61)	.69
Male sex	77 (55%)	39 (45%)	.22
BMI	24 (21-27)	24 (21-27)	.73
LuTx indication			.34
COPD	53 (38%)	32 (37%)	
ILD	61 (43%)	41 (48%)	
CF	20 (14%)	13 (15%)	
PH	6 (4%)	0 (0%)	
BE	1 (1%)	0 (0%)	
HU status	36 (26%)	28 (33%)	.32
Waiting list time, y	0.8 (0.2-2.1)	1.1 (0.4-2.3)	.26
Hospitalization pre-Tx	43 (31%)	26 (30%)	1.00
ICU pre-Tx	18 (13%)	10 (12%)	.96
ECMO pre-Tx			.77
VV	6 (4%)	5 (6%)	
VA	5 (4%)	2 (2%)	
VAV	1 (1%)	0 (0%)	
No ECMO	129 (92%)	79 (92%)	

Continuous variables are presented as median (interquartile range). Categorical variables are presented as n (%). *BMI*, Body mass index; *LuTx*, lung transplantation; *COPD*, chronic obstructive pulmonary disease; *ILD*, interstitial lung disease; *CF*, cystic fibrosis; *PH*, pulmonary hypertension; *BE*, bronchiectasis; *HU*, high urgency; *ICU*, intensive care unit; *Tx*, transplantation; *ECMO*, extracorporeal membrane oxygenation; *VV*, venovenous; *VA*, venoarterial; *VAV*, venoarterial-venous.

**Table E2:** Unadjusted (univariable model) and adjusted (multivariable model) estimates for the effect of time of surgery (operating during the day as reference) on the outcome

Outcome	Univariable		Multivariable	
	β/IRR (95% CI)	*P* value	β/IRR (95% CI)	*P* value
LOS ICU, d∗	0.78 (0.52-1.15)	.22	0.80 (0.54-1.17)	.25
LOS hospital, d∗	1.00 (0.79-1.25)	.97	1.00 (0.80-1.25)	.99
Time to extubation, d∗	0.62 (0.31-1.16)	.16	0.63 (0.33-1.14)	.14
Operation duration, min, log transformed†	−0.11 (−0.19 to −0.04)	.004	−0.08 (−0.15 to −0.02)	.01

In the multivariable model, outcomes were corrected for age, sex, body mass index, high urgency status, lung transplantation indication, intraoperative extracorporeal circulation use, and operative technique. *IRR*, Incidence rate ratio; *LOS*, length of stay; *ICU*, intensive care unit.

∗ Quasi-Poisson regression with incidence rate ratio.

† Linear regression with β (transformation in first column).
